# Introduction to “Five Years of the Sendai Framework for Disaster Risk Reduction”

**DOI:** 10.1007/s13753-020-00271-0

**Published:** 2020-04-15

**Authors:** Ilan Kelman

**Affiliations:** 1 Institute for Risk & Disaster Reduction and Institute for Global Health, University College London, London, WC1E 6BT UK; 2 University of Agder, Kristiansand, Norway

Are birthdays important, especially for documents? While some might use the candles on the cake as an excuse to burn the offending paper, they also offer a chance to reflect on any milestones or achievements that were hoped for while examining possible futures based on guidance from the document. Thus was born the idea of this special issue on “Five Years of the Sendai Framework for Disaster Risk Reduction.”

This special issue also follows directly from the June 2015 issue of this journal (Vol. 6, No. 2) devoted to analyzing the then-new agreement, bringing hope to the optimists and sardonicism to those decrying yet one more international document that might or might not yield needed action—or distract from it. The Sendai Framework is indeed one more international document for disaster risk reduction, after the Hyogo Framework for Action 2005–2015 that followed the International Decade for Natural Disaster Reduction (1990–2000), which followed many predecessors. It promotes a sustainability-focused approach, along with several others also signed in 2015 including the Sustainable Development Goals and the Paris Agreement on climate change. All feed into an agenda towards 2030 by which time it might be too late to do anything useful.

Or perhaps the Sendai Framework is yet one more international document to galvanize needed action and to hold national governments to account on a short timeframe. Whether or not national governments actually have the power to effect the change we need is another question.

So which is it? Good, bad, ugly, beautiful, wise, silly, or a combination? The reader can decide based on the contributions to this special issue.

First, we are privileged to have varying perspectives from the current and former Special Representatives of the United Nations Secretary-General for Disaster Risk Reduction—the heads of the UN agency responsible for disaster risk reduction, which was UNISDR (United Nations International Strategy for Disaster Reduction) and is now UNDRR (United Nations Office for Disaster Risk Reduction). The current Special Representative, Mami Mizutori, provides a detailed and balanced perspective of what the Sendai Framework proffers and what it might yet achieve, while her predecessor, Robert Glasser, focuses on climate change.

Next, a quartet of powerful papers offers case studies of people and places for Sendai + 5. DeeDee Bennett highlights people with disabilities, not just what they get and do not get from the Sendai Framework, but also what they can and should provide. Elizabeth Maly and Anawat Suppasri examine the relevance of the Sendai Framework to Japan following the 11 March 2011 disaster, focusing on tsunami engineering and disaster recovery aimed at reducing risk. Supplementing this disaster-based perspective, Dewald van Niekerk et al. and Wendy Saunders et al. explore Africa and New Zealand respectively, to provide continent-wide and national analyses.

This special issue next moves into three cross-cutting papers. Natalie Wright et al. assembled an impressive team heavily involved in the Covid-19 epidemic and then pandemic as they were writing the article. They look at the relevance of health and disaster risk reduction for each other in terms of the Sendai Framework. Meanwhile, Victor Marchezini highlights warning systems, particularly influenced by different disciplinary perspectives and framed by his own experiences. Next, Marie Aronsson-Storrier gives an astute critiquing analysis of the position of the Sendai Framework with respect to international law.

All these pieces are anchored by the concluding contribution from Ben Wisner posing and answering a slew of questions that he had asked and analyzed 15 years previously for the Sendai Framework’s predecessor, the Hyogo Framework for Action. The special issue therefore circles back to Mizutori’s opener by providing an agenda for boosting the Sendai Framework’s successes while pushing forward with areas for improvement.

The result of combining these authors, ideas, and articles—as expected—provides no firm conclusions. Instead, it is a mixed bag of advantages, disadvantages, neutral traits, missed opportunities, and bold leadership that is saving lives daily and decadally. National governments can and do act—and sometimes they cannot and do not act. The same for people, nonnational governments, intergovernmental agencies, nonprofit groups, and for-profit organizations. Ultimately, the key challenge is to find commonalities for pushing forward together and collaboratively for common goals, irrespective of a diversity of resources, interests, and pathways.

This also means identifying and filling in the gaps. This special issue includes authors living on every inhabited continent with a balance of ages, career stages, disciplines, ethnicities, and genders. But who is missing? I did not know or enquire about other characteristics such as disability, sexuality, identity, and citizenship. Similarly, so many key topics are absent, such as gender, Arctic experiences, islander perspectives, mountain peoples, local and indigenous knowledges and wisdoms, everyday risks, prisoners, homeless people, and the roles of specific hazard influencers such as climate change, El Niño, and multi-decadal oscillations. There is plenty more to do in terms of learning from, connecting with, and improving the Sendai Framework.

Does this collection inspire you with hope that so much has been and is being achieved? Or is it despairing how far there is yet to go and the machinations and convolutions required to arrive there? Or is it the standard melange of conflicting emotions? Hopefully, at minimum, we will be contemplating what each of us can offer and do while munching on the Sendai Framework’s fifth birthday cake.

